# Hospitals and Their Special Needs

**Published:** 1917-06-23

**Authors:** 


### HOSPITALS AND THEIR SPECIAL NEEDS.

**Acton Hospital.**—Since its foundation in 1898 the hospital has admitted 3,966 in-patients and attended and treated 21,288 out-patients, 4,455 district nursing cases, and 5,568 dispensary patients. Funds for maintenance are asked for.

**Baldwin Brown Home, Herne Bay.**—Despite "flying visits of the enemy to the town and neighbourhood," the thirty beds of this home are used with much appreciation by the convalescent poor.

**Beckenham Cottage Hospital.**—The special need this year is help towards the cost of repairs, restoration, and improvements to the buildings.

**Belgrave Hospital for Children, Clapham Road, S.W.** The Belgrave Hospital for Children is the only hospital for children in South-West London, and is situated in the midst of a densely populated neighbourhood of working people. In 1916 686 children were admitted, of whom 199 were under one year of age, and 37,968 attendances were made in the out-patient department. In view of the serious infantile mortality in this country, these figures bear eloquent testimony to the effort which is being made at this hospital to save the lives of the infants.

**Brentwood Convalescent Home.**—This home is fo London children, and the demand on the beds is greater than ever, as so many homes have been taken over for military purposes. Funds are much needed for household expenses.

**British Hospital for Mothers and Babies, and National Training School for District Midwives, Woolwich.**—In view of the present importance of infant life, the hospital is making a supreme effort to collect the sum needed for building, so that there may be no unnecessary delay at the close of the war.

**Canning Town Women's Settlement Hospital, Plaistow, E.**—With the larger hospitals crowded, additional responsibilities are thrown upon the smaller ones. The sick poor to whom this hospital ministers in Canning Town have more claim upon the giving public now than they have ever had.

**Central London Throat, Nose, and Ear Hospital Gray's Inn Road.**—Twenty of the forty-two bed are given over entirely for soldiers, who are sent by the military authorities for that treatment for which the hospital is specially equipped. The sick civil poor have not been deprived of the accommodation made for their benefit. Funds are diminished, and help is asked.

**Charing Cross Hospital.**—The hospital has succeeded in reducing the mortgage debt from its original large amount of £85,000 to £22,000, and wants to clear this off by getting 2,200 contributors of £10 each. When this has been done the hospital will be in a position of absolute safety, as those who are supporting it are providing sufficient funds to keep the hospital going on its enlarged basis of 300 beds.

**Chelsea Hospital for Women, Fulham Road, S.W.**—It is estimated that the sum of about £23,000 is still required to enable the committee to complete the rebuilding scheme. Special facilities are being given for the treatment of wives and other near relatives of soldiers at the Front.

**Children's Hospital for the Treatment of Hip Disease, Sevenoaks.**—The hospital is greatly in need of help. The ever-increasing cost of all necessities causes a great strain on the management, and it is feared that without further public generosity there will be a heavy deficit at the end of the year. The hospital is now recognised under the Local Government Board as a hospital for the treatment of surgical tuberculosis for children under school age. There is a great demand for the beds at present.

**City of London Hospital for Diseases of the Chest, Victoria Park, E.**—For the third year in succession the Committee of Management have felt obliged, in consequence of the war, to give up the annual festival dinner, which has always proved a valuable means of obtaining help for the hospital, and are, therefore, constrained to appeal for contributions as widely as possible. It is hardly necessary to say that the rise in the prices of nearly all the things that a hospital needs—food and drugs in particular—adds very greatly to the expenses; at the present time the extra cost in the case of this hospital is not less than £60 a week.

**City of London Lying-in Hospital.**—At the present time the work of a lying-in hospital is particularly important, and the value of an institution which helps to save the infant life of the nation, and so to provide the men and women of to-morrow, cannot be too strongly emphasised. This hospital receives a great number of the wives of our sailors and soldiers.

**East End Mothers' Lying-in Home, Commercial Road, E.**—To carry on the work of the home new subscriptions are wanted. The greater portion of the work is now being done among the wives of sailors and soldiers.

**East London Hospital for Children, Shadwell, E.**—Reduced incomes and money diverted to war funds have materially affected the funds of this hospital, while the enormously increased cost of all commodities has much accentuated the Board's difficulties. Many children are treated whose fathers are fighting at the Front, and who come to see them when on furlough.

**Eversfield Chest Hospital, West Hill Road, St. Leonards-on-Sea.**—Annual subscriptions and donations are needed to maintain the hospital in its present state of efficiency. A sum of £660 is required to carry to completion the cliff wall that it has been necessary to erect to safeguard the hospital buildings, which were being menaced by the serious crumbling of the cliff bounding the grounds on the south side. A further sum of £4,300 is also needed to pay off the mortgage.

**French Hospital and Dispensary, Shaftesbury Avenue.**—This hospital is open to all foreigners speaking French, and in 1916 802 patients were admitted. The hospital placed thirty beds at the disposal of the British War Office and Admiralty, and the French Convalescent Home at Brighton sixty. Over 1,400 British wounded have been received both at the hospital and the convalescent home from the beginning of the war.

**Gordon Hospital, Vauxhall Bridge Road, S.W. 1.**—The chief need is help in respect of a heavy mortgage debt of £3,700. Relieved of this burden, the anxiety of the committee of management would be greatly curtailed.

**Grosvenor Hospital for Women, Vincent Square, S.W. 1.**—Starting the year with a deficit of £630, the reduction of income still continuing, the hospital is

[*Continued on p.* 236.

### Hospitals and their Special Needs.

[Continued from p. 233.

in special need of funds at this time. A thorough reorganisation of the heating arrangements throughout the building has become necessary, involving the hospital in heavy capital expenditure, and the varying and increasing cost of all provisions and necessaries is causing the greatest anxiety to the committee. Contributions will be gladly received by the Secretary, Mr. W. J. Davidson.

**Guy's Hospital, S.E.**—It is obvious that the prolongation of the war, affecting all phases of our national life, lays a heavy burden upon hospitals and those responsible for their administration. The strain is felt mainly in two directions: the shortage of staff and the cost of supplies; and in both it tends constantly to become more acute. The Governors of Guy's Hospital have much cause for thankfulness that in a time of such heavy financial demands the income has shown a capacity to expand to the hospital's needs, mainly attributable to the fact that the receipts from legacies have recently been much above the average. This augmented income, however, has partly had to be used to meet additional expenditure, leaving very little in hand for future contingencies. These are bound to arise, and in face of such facts it is plain that friends of the hospital ought not to relax their financial support. The endowments of the hospital, though considerable, are far from being sufficient to cope with the volume of work with which Guy's has to deal. Serious, indeed, would be the position were the Governors unable to rely upon a generous measure of private benevolence. It is as true as ever that "Guy's Hospital needs help," and, as its record shows, deserves it.

**Hampstead General and North-West London Hospital.**—The development of the hospital since the amalgamation has been more rapid than the expansion of its income, and the total indebtedness of the institution is now £5,000. The Council consequently make an earnest appeal for new annual subscriptions and donations. Over 600 wounded soldiers have been treated, and fifty-three beds are held at the disposal of the War Office.

**Hospital for Consumption, Brompton, S.W.**—Including the sanatorium at Frimley the hospital has about 500 beds constantly occupied. Many sailors and soldiers have been admitted since the outbreak of the war. Special arrangements have also been made for the admission of dependants of sailors and soldiers serving with H.M. Forces if needing treatment. Money is the one essential required, and the committee appeal to the generous and patriotic public to come to their aid with funds sufficient to carry on the work.

**Hospital for Sick Children, Great Ormond Street, W.C.**—It is anticipated that, unless the unexpected happens during the year 1917, a deficit between assured income and expenditure of about £5,000 will have to be met to keep the hospital out of debt. The need for greater effort to counterbalance the drain of war upon the manhood of the nation, by saving infant life for the future welfare of the British Empire, compels the committee to plead most earnestly for continued and increased support for the national work this hospital is performing in the preservation of child-life.

**Hospital for Women, Soho Square, W.**—During 1916 1,035 patients were admitted, and 12,638 patients attended in the out-patient department, while there were usually 100 patients or more on the waiting list for admission. New annual subscribers are wanted to help to reduce the mortgage debt, which now stands at £4,000, and costs the hospital £200 a year for interest.

**Hounslow Hospital.**—An appeal is made for an increase in regular annual support, which is not growing at the same rate as the expenditure.

**Infants Hospital, Vincent Square, Westminster, S.W.**—An extra £1,800 must be raised to meet the expense of maintenance for this year. Besides carrying on the usual work of the hospital, which is now, if possible, more important than ever, the committee has provided free accommodation for war nurses.

**King Edward Memorial Hospital, Ealing.**—Of the seventy beds twenty are temporary and are used for soldiers. The expenditure has increased by 15 per cent. since 1915.

**King's College Hospital, Denmark Hill, S.E.**—The removal of the hospital from the Strand district to South London has unfortunately had a serious effect on its finances. It has necessarily lost its local connection, and is only gradually building up a new one in South London. It is hoped, however, that residents in this district will liberally respond, for the hospital is in urgent need of support for its ordinary work, and is still heavily burdened by a large debt on the new buildings, which have now all been taken into use. On the outbreak of the war a considerable portion of the hospital was placed at the disposal of the War Office for the use of the 4th London General Hospital, retaining the casualty department, and at least four wards for the use of the civilian population. An appeal is being made for £100,000 to pay off the debt on the building fund, and to provide for present and future requirements.

**London Homœopathic Hospital, Great Ormond Street, W.C.**—To provide the sum to be raised by December 31 of the current year to pay off debts to capital, £16,710, unavoidably incurred in the maintenance of the hospital, many generous donations are necessary. Over 500 wounded sailors have been treated to date.

**London Hospital, Whitechapel, E.**—The work of the London Hospital, like that of all other voluntary hospitals, is carried on with great difficulty. Shortage of staff, both medical and lay, has necessitated considerable reorganisation in the hospital's work, although every endeavour is made to prevent unnecessary suffering and inconvenience to the sick poor. For the moment the hospital is not taking in wounded men, but has promised to do so as soon as the pressure on the War Office becomes acute. During the time that some of its beds were lent to the War Office nearly 4,000 wounded men were treated. Two floors are at present set aside for the use of wounded and sick officers, of whom 881 have so far been treated. The difficulty of procuring provisions of all kinds continues, and, as every housekeeper knows, the prices of coal, milk, bread, meat, vegetables, and grocery have all advanced. In addition to these, a hospital has to buy many things which the housekeeper does not need—bandages, lint, cotton-wool, instruments for the operating theatre, etc. All these have advanced in price, just as provisions have. It may be truly said that never before have hospitals had to pass through such a difficult period.

**London Temperance Hospital, Hampstead Road, W.** This hospital was founded in 1873 to demonstrate the possibility of successfully treating disease and personal injuries without (at the time) universal use of alcohol. It is a general public hospital, with 116 available beds, and out-patient and casualty departments. During the past year 182 soldiers, sick or wounded, were received as in-patients. The board feel confident that a larger knowledge of the work done there will result in much greater financial support to meet the increase of cost of maintenance.

**Metropolitan Ear, Nose, and Throat Hospital, Fitzroy Square, W.**—When the war broke out this hospital was placed at the disposal of the naval and military authorities. Beds were set apart, and have been continuously occupied by the wounded, without precluding at the same time adequate treatment of civilian patients. The work lately has been a great strain on the finances of the hospital, and donations and subscriptions are urgently needed.

**Metropolitan Hospital, Kingsland Road, N.E.**—The recent air raids have confirmed the committee in the wisdom of continuing ample provision for the civilian population. Indeed, the circumstances of the district and its population absolutely compel it. Attached to the hospital there was opened, in October 1915, the adjacent L.C.C. Enfield Road School; accommodation for 302 sick and wounded soldiers has thus been provided. The Metropolitan Hospital at the present time is in serious need of help. For those who cannot send money, gifts of clothing, food, fruit, eggs, etc., and flowers are very acceptable.

**Miller General Hospital for South-East London, Greenwich Road, S.W.**—Contributions are needed towards making good the deficiency in income which will be caused in this year's accounts by the war. The work of the hospital has been carried on, but not without difficulty and much anxiety in regard to funds. The annual cost is about £10,000, and the assured income (from investments) is only £613. A debt of £4,581 still remains unpaid on the new wing, and the imperative rebuilding of the nurses' home and the out-patient department at a cost of at least £20,000 must be put in hand immediately after the war. A ward has been set apart for wounded, and this has been continuously occupied. The hospital needs of the civilian population have not been lost sight of, and many more beds have been occupied by them than before the war.

**Mount Vernon Hospital for Consumption and Diseases of the Chest, Northwood.**—Provision is made for those who are excluded from the benefits of the Insurance Act, and special provision is made for throat and heart cases and for children. The hospital is delightfully situated amid charming surroundings. Funds are urgently needed to continue the good work. At the moment the committee are in debt to their bankers.

**National Dental Hospital (Dental Department of University College Hospital), Great Portland Street.**—The numbers of ordinary patients have increased, and among these there is a continuous increase in the number who have to be treated quite free, and a decrease in the number who can afford to pay the very small charges asked from those who are unable to pay a dentist's charges.

**National Hospital for Paralysed and Epileptic, Queen Square, W.C.**—An appeal is made for new annual subscriptions and donations to meet the increased cost of maintenance which in many ways has particularly affected this institution, which has to purchase expensive drugs, etc., on a large scale. There are seventy beds for soldiers suffering from nerve injuries and severe mental and nervous shock. Every effort is being made to provide accommodation for the needs of the civil public, and extra beds have been temporarily added.

**North London or University College Hospital, W.C.** The total number of beds has been temporarily increased to 492, 250 of which are allotted to sailors and soldiers. The total number of in-patients treated in the wards was 5,475, of whom over 1,700 were wounded sailors and soldiers. The total number of out-patients treated in the various departments was 52,880. New features of the hospital are the dental department, the maternity and child welfare department, and the tuberculosis dispensary. The hospital is almost entirely dependent on voluntary contributions.

**Paddington Green Children's Hospital, W.**—This hospital affords gratuitous medical and surgical relief to sick children of the poor. The average ordinary yearly income of the hospital and home falls short of the expenditure by about £1,000. The committee urgently appeal for new annual subscriptions and donations.

**Passmore Edwards Hospital for Willesden, Harlesden Road, N.W.**—There are now 70 beds instead of twenty-five, and funds are much wanted to enable this hospital to treat its soldier and civilian patients and to pay for an *x*-ray department recently erected.

**Passmore Edwards Hospital, New Southgate.**—A new system of steam heating has been decided upon, and contributions towards the cost of installation are asked for.

**Poplar Hospital for Accidents, East India Dock Road, Poplar, E.**—Funds are needed to enable the hospital to continue to pay its way. Patients are treated at the rate of six per hour, day and night, and for sixty years the hospital has been free from debt, but the great increase in food, drugs, and dressings, and increased salaries, will make our task very much harder. Twenty-four beds have been set aside for wounded soldiers, but only twelve have been withdrawn from our civilian patients. A large number of the victims of the great explosion in the East End were treated at the hospital.

**Queen Charlotte's Lying-in Hospital, Marylebone Road, N.W.**—The hospital is in great need of funds for maintenance, being in debt to the extent of £5,000. Since the outbreak of war over 4,000 wives of our sailors and soldiers have been admitted to the wards or attended and nursed at home, as well as many Belgian and other refugees. Further contributions are earnestly solicited.

[*Continued on p.* 242.

### Hospitals and their Special Needs.

[Continued from p. 237.

**Reigate and Redhill General Hospital.**—The chief need is modestly stated to be a modern invalid wheel-chair with a leg rest and on two big wheels only. A good chair would be a real boon for the bad cases.

**Royal Dental Hospital, Leicester Square, W.C. 2.**—There has been a heavy decrease in annual subscriptions, and a very earnest appeal is made for new subscriptions and life governors' and other donations.

**Royal Eye Hospital, Southwark, S.E.**—The Royal Eye Hospital is the only ophthalmic hospital in South London, and draws its patients from a very wide area. The number of new out-patients increased from 21,949 to 22,715; this is almost entirely the outcome of the large numbers of men and women engaged in munition work in the neighbourhood, accidents from machinery and cases of eye-strain being very numerous. 626 in-patients were treated, including fifty-nine sailors and soldiers. The Council appeal to all well-wishers of the charity to assist them in the beneficent work by contributing themselves and by making its needs known amongst their friends.

**Royal Free Hospital, Gray's Inn Road, W.C.**—2,484 patients were admitted to the wards and 115,913 attendances of out-patients are recorded for the past year. On behalf of the Country's Charge Fund for further providing for infant welfare an appeal for £200,000 is made. This will enable the hospital authorities to provide ante-natal and infant welfare wards, as well as accommodation for tuberculous and special cases, on a valuable site adjoining the hospital and presented to it for this purpose.

**Royal Hospital for Diseases of the Chest, City Road.** This institution has been in existence for over a century, and was the first in Europe to specialise in chest diseases. Its admirable work is less well known than it deserves, for, situate, as the hospital is, in one of the poorer and more distant parts of London, the wealthy do not often pass its doors. But it is to be hoped that those who can help will not pass it by. The very fact that it is among the poor, and for the poor, adds to the value of its work and the strength of its claim.

**Royal London Ophthalmic Hospital (Moorfields Eye Hospital), City Road, E.C.**—One physician, one surgeon, one assistant surgeon, the *x*-ray officer, the bacteriologist, twelve chief clinical assistants, and four clinical assistants are serving with the Forces. In spite of this the work has been carried on, but the strain is severe. Last year there were 2,396 in-patients and 47,438 new out-patients; and the total number of attendances was 124,095. By arrangement with the military authorities thirty beds are set aside for wounded soldiers requiring ophthalmic treatment. The urgent need of the hospital is an extension with rooms for nurses, for whom the present accommodation is inadequate.

**Royal National Sanatorium for Consumption, etc., Bournemouth.**—It shows the widespread usefulness of this institution that of the patients admitted in 1916, 420 came from no fewer than thirty-one different counties. Many of the patients were sailors and soldiers. The annual subscriptions show a continued falling off, while expenditure has heavily increased.

**Royal National Hospital for Consumption, Ventnor.** Funds are urgently needed for the maintenance of the large number of patients. The institution enters upon the fiftieth anniversary of its foundation this year. The annual expenses exceed £16,000, being £2,500 over 1914, by reason of the general increase in prices occasioned by the war. A number of sailors and soldiers suffering from consumption are now under treatment at this hospital.

**St. Andrew's Convalescent Home, Clewer.**—An appeal is made to all housekeepers, who will specially appreciate the difficulties of a hospital in these days. There is "an uncomfortably small sum in hand with which to meet current expenses."

**St. George's Hospital, S.W. 1.**—In 1916 4,657 in-patients and 27,471 out-patients received treatment at this hospital, and of the former 925 were sent to the convalescent branch at Wimbledon at no cost to themselves. Whilst ministering to the sick poor as usual, a number of beds have been allocated for the reception and treatment of sick and wounded sailors and soldiers, of whom over 1,400 have now been treated. The house committee most urgently appeal for contributions to enable them to reduce the deficiency with which they are faced during the present year.

**St. John's Hospital for Diseases of the Skin, 49 Leicester Square, W.C. 2.**—Secretary, George A. Arnaudin.—For fifty-four years St. John's has been doing steady and excellent work, and at the present time some 300 in-patients are treated annually, and in round figures 8,000 out-patients, making a total of about 40,000 attendances. Special help is needed for renewing its electrical plant—cost, about £250. The hospital is treating venereal diseases under the Government scheme.

**St. John's Hospital, Morden Hill, Lewisham, S.E.**—The number of beds was increased to 62 during the past year; 482 in-patients were treated, of whom 165 were soldiers. The number of attendances on out-patients—chiefly confined to those requiring surgical, bacteriological, or *x*-ray treatment—was 11,151, as against 9,164 in 1915. Funds are urgently needed to enlarge the out-patients' department, the present accommodation being quite inadequate.

**St. Mark's Hospital, City Road, E.C.**—Before the war the committee had decided, at a cost of over £1,000, to build a special women's ward for cancer patients, also additional accommodation for the nursing staff, but unless the charitable public see their way to support this effort to cope with the ever-increasing cases of rectal cancer this scheme must become at present impossible. Twelve beds have been reserved for wounded soldiers.

**St. Monica's Home Hospital for Sick Children, 16 Brondesbury Park, N.W.**—For over forty year this hospital has carried on its beneficent work, and until the past two years free from debt. Recently, however, there has been a considerable falling-off in the receipts, from donations chiefly, and at the end of 1916 there was a deficiency of £310.

**St. Mary's Hospital, Paddington, W.**—This hospital has 305 beds, and its work costs £35,000 a year. St. Mary's has manufactured and supplied not only our own but all the Allied Armies with anti-typhoid and other vaccines. Several million doses have been supplied, and this work has been acknowledged by the Army Council as "of great practical value to the troops in the field as well as to the State." Similar testimony has been borne by the Belgian, French,

[Continued on p. xvi.

